# Delayed and repeated intranasal delivery of bone marrow stromal cells increases regeneration and functional recovery after ischemic stroke in mice

**DOI:** 10.1186/s12868-018-0418-z

**Published:** 2018-04-12

**Authors:** Monica J. Chau, Todd C. Deveau, Xiaohuan Gu, Yo Sup Kim, Yun Xu, Shan Ping Yu, Ling Wei

**Affiliations:** 1 Department of Anesthesiology, Emory University School of Medicine, Atlanta, GA 30322 USA; 2 Department of Neurology, Emory University School of Medicine, Atlanta, GA 30322 USA; 3 Department of Neurology, Nanjing University School of Medicine, Nanjing, China; 4 Center for Visual and Neurocognitive Rehabilitation, Veteran’s Affair Medical Center, Atlanta, GA USA; 5 Woodruff Memorial Research Building, Suite 617, Emory University School of Medicine, 101 Woodruff Circle, Atlanta, GA 30322 USA

**Keywords:** Ischemic stroke, BMSC, Intranasal, Hypoxic preconditioning, Trophic factors

## Abstract

**Background:**

Stroke is a leading cause of death and disability worldwide, yet there are limited treatments available. Intranasal administration is a novel non-invasive strategy to deliver cell therapy into the brain. Cells delivered via the intranasal route can migrate from the nasal mucosa to the ischemic infarct and show acute neuroprotection as well as functional benefits. However, there is little information about the regenerative effects of this transplantation method in the delayed phase of stroke. We hypothesized that repeated intranasal deliveries of bone marrow stromal cells (BMSCs) would be feasible and could enhance delayed neurovascular repair and functional recovery after ischemic stroke.

**Results:**

Reverse transcription polymerase chain reaction and immunocytochemistry were performed to analyze the expression of regenerative factors including SDF-1α, CXCR4, VEGF and FAK in BMSCs. Ischemic stroke targeting the somatosensory cortex was induced in adult C57BL/6 mice by permanently occluding the right middle cerebral artery and temporarily occluding both common carotid arteries. Hypoxic preconditioned (HP) BMSCs (HP-BMSCs) with increased expression of surviving factors HIF-1α and Bcl-xl (1 × 10^6^ cells/100 μl per mouse) or cell media were administered intranasally at 3, 4, 5, and 6 days after stroke. Mice received daily BrdU (50 mg/kg) injections until sacrifice. BMSCs were prelabeled with Hoechst 33342 and detected within the peri-infarct area 6 and 24 h after transplantation. In immunohistochemical staining, significant increases in NeuN/BrdU and Glut-1/BrdU double positive cells were seen in stroke mice received HP-BMSCs compared to those received regular BMSCs. HP-BMSC transplantation significantly increased local cerebral blood flow and improved performance in the adhesive removal test.

**Conclusions:**

This study suggests that delayed and repeated intranasal deliveries of HP-treated BMSCs is an effective treatment to encourage regeneration after stroke.

## Background

Stroke is a leading cause of death and disability worldwide with only one FDA-approved drug treatment, tissue plasminogen activator (tPA), in the U.S. [1]. tPA is a thrombolytic agent that may show therapeutic effect acutely after stroke; delayed administration of tPA after its therapeutic window of 4.5 h has increased risk of hemorrhagic conversion [2]. Additionally, a set of exclusion criteria precludes many patients from receiving the tPA treatment regardless of the time window. These may include uncontrolled hypertension, indication of intracranial hemorrhage, seizure at the onset of stroke, and a history of arteriovenous malformation or aneurysm. As another impedance, patients that were asleep during the onset of stroke cannot accurately pinpoint the time of occurrence. Thus, it appears necessary and important to develop delayed treatments several hours or even several days after stroke.

Transplantation of cells such as bone marrow stromal and bone marrow stem cells (BMSC) has been explored as a regenerative avenue for stroke therapy [3–5]. The regenerative phase is thought to have a wide therapeutic window. Cells can be transplanted in the delayed phase of stroke, from days to a month after stroke [6, 7]. BMSCs are already clinically used for therapy in autologous and allogeneic transplantation for diseases such as leukemia and sickle cell anemia [8, 9]. Similar to other types of stem and progenitor cells, BMSCs produce trophic factors that are beneficial for the recovery of brain damage. For example, BMSC-conditioned media enhances neurite outgrowth and neurite length in Ntera-2 neurons, demonstrating the paracrine effects of BMSCs in vitro [10]. Adaptive factors released by mesenchymal stem cells include cytoprotective factors (endothelin-1), angiogenic factors (VEGF, Smad4, and Smad7), and pro-migration factors (LRP-1, LRP-6) [11]. Previous studies showed that trophic factors secreted by transplanted BMSCs contributed to tissue repair, ultimately leading to improved functional recovery [12, 13]. An intravenous infusion of BMSC- and BMSC-conditioned media led to neurogenesis and an attenuation of macrophage/microglia invasion in the brains of ischemic stroke mice [14]. Although the exact mechanism remains to be further identified, it is suggested that transplanted BMSCs can serve as vehicles for regenerative and anti-apoptotic factor delivery through their paracrine actions after administration [11].

Endogenous regeneration occurs in the adult mammalian brain through processes such as neurogenesis in which neural progenitor cells are continuously generated in regenerative niches such as the subventricular zone (SVZ). Following ischemic injury, neuroblasts are diverted from the rostral migratory stream toward the ischemic region by chemoattractive factors specifically the stromal cell-derived factor-1 α (SDF-1α) [15–17]. This response appears to be an attempt at self-repair after a CNS injury. However, it is estimated that as many as 80% of SVZ-derived new neurons at the ischemic site die 6 weeks post-ischemia, possibly due to the detrimental cytotoxic effects of the injured environment [18]. Treatments aimed at bolstering this endogenous repair may prove to be a promising strategy for stroke therapy.

There are currently several methods of stem cell delivery for brain disorders: intravenous/intra-arterial infusion, intracerebral injection, and more recently, intranasal administration. Previous studies with BMSC transplantation focused on intracerebral and intravascular routes [19–21]. Even though these studies demonstrate the therapeutic potential of BMSCs after stroke, these delivery methods are either invasive or inefficient. For example, intracranial administration requires a craniotomy surgery and cell injection with a needle or a pipette that can damage brain tissue. Cells delivered systemically have low homing rates to the brain; the majority of cells are found in peripheral organs primarily in the lungs, liver, and spleen following delivery [22, 23].

As a way to bolster transplantation efficacy, our group has reported that hypoxic preconditioning (HP) of BMSCs and neural progenitor cells conveys several benefits [3, 24, 25]. HP increases the survival of transplanted cells in the ischemic heart and brain [3, 25], enhances BMSC homing capacities to the brain infarct region through increased expression of the SDF-1 ligand CXCR4 [3]. Intranasally delivered BMSCs in the acute phase of stroke reduced the volume of the ischemic infarct, decreased the number of TUNEL-positive cells in the peri-infarct region, and improved sensorimotor functional recovery of after stroke [3]. In the present investigation, we aimed to demonstrate that the time window for intranasal administration of BMSCs can be extended past 24 h after stroke. Taken the advantage of the non-invasive nature of the nasal route, we demonstrated that delayed and repeated administrations of BMSCs could be applied for enhancing endogenous regenerative activities and sustainable functional recovery after stroke.

## Methods

**Fig. 1:**
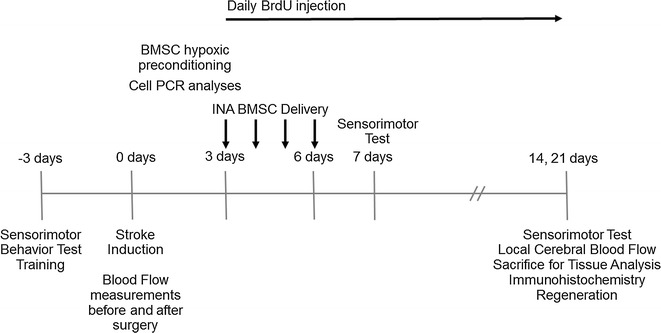
Experimental timeline. Focal ischemic stroke was induced in adult male mice on “day 0”. Three days after stroke, a total volume of 100 μl of BMSC suspension (~ 1 × 10^6^ cells) or control media was administered via the intranasal route at 3, 4, 5, and 6 days after stroke. Starting on the same day as BMSC administration, all mice received daily BrdU injections intraperitoneally (i.p) until the day of euthanasia. Laser-Doppler scanning was performed 21 days after stroke to measure changes in the local blood flow in penumbra. The adhesive removal test of sensorimotor function was performed at 7 and 14 days after stroke. Immunohistochemistry was performed at 14 days after stroke to analyze regeneration

The experimental timeline is summarized in Fig. 1.

### BMSC cell culture

BMSCs were isolated and cultured as previously described [3, 24]. Briefly, GFP-expressing BMSCs were dissected from the tibias of postnatal day 21 transgenic Wistar rats (Charles River Laboratories, Wilmington, MA). Cells were cultured and maintained in DMEM media with 15% BMSC Fetal Bovine Serum (FBS) and 1% Penicillin–Streptomycin to prevent contamination of cell culture. Cells were trypsinized with 0.25% trypsin–EDTA and then deactivated with 15% FBS media before being plated into dishes. After 24 h, non-adherent cells were removed and fresh medium was added to adherent cells. Upon isolation, the BMSC population was characterized via fluorescence-activated cell sorting using cell surface markers CD34, CD45, CD73, CD90, and CD105 (eBioscience, San Diego, CA or BD Pharmingen, Rockville, MD). All cells used in this study were freshly isolated, used at low passages (within 4 passages) when they were 80-90% confluent, and maintained at normoxic oxygen until hypoxic preconditioning.

### Immunocytochemistry

Cells in 3.5 cm tissue culture dishes were fixed with 4% paraformaldehyde for 10 min. The dishes of cells were washed with 1× phosphate buffered saline (PBS) 3 times for 5 min per wash. Ethanol:acetic acid (2:1) was applied for 10 min and washed with PBS. 0.2% Triton-X 100 was applied for 10 min for cell permeabilization and washed out with PBS (3 times, 5 min each). The cells were blocked with 1% cold fish gelatin (Sigma, St. Louis, MO) for 1 h and primary antibodies were applied at a concentration of 1:100 overnight at 4 °C for SDF-1α (MAB350, R&D Systems, Minneapolis, MN), CXCR4 (R&D Systems), VEGF (Millipore, Billerica, MA) and FAK (c-20; Santa Cruz Biotechnology). A secondary antibody (Jackon ImmunoResearch, West Grove, PA) corresponding to the host animal of the primary antibody was applied and incubated at room temperature for 1 h then washed with PBS. Hoechst 33342 was applied at a concentration of 1:25,000 for 5 min and washed with PBS. Dishes were cover-slipped with Vectashield mounting media (Vector labs, Burlingame, CA). Photographs were taken with a fluorescent microscope (BX51, Olympus, Tokyo, Japan).

### Hypoxic preconditioning of BMSCs

BMSCs (70–90% confluent) were incubated for 24 h in the ProOx-C-chamber system (Biospherix, Redfield, NY) at 0.1–0.3% oxygen. After 24 h, the cells were returned to normoxic, culture conditions for 1 h of reoxygenation. Following reoxygenation, cells were either harvested for PCR analysis, fixed for immunohistochemistry, or harvested for intranasal delivery (Fig. 1). Only HP-treated BMSCs were used for transplantation in this investigation; comparisons between HP-BMSCs and non-HP BMSCs have been established where HP benefits were great enough for us to continue the use of HP on BMSC as standard protocol [3].

### PCR analysis

mRNA was harvested from the control BMSC and HP-BMSC culture dishes using Trizol reagent (Invitrogen Life Technologies, Grand Island, New York). 250 μl of Trizol was used per 3.5 cm dish of BMSC. The cells were scraped in Trizol and collected, vortexed, and briefly incubated for 5 min to allow for the full dissociation of nucleoprotein complexes. Fifty μl of choloroform was added to the mixture to separate RNA into a colorless phase from the mixture. This colorless aqueous phase was separated into a new tube. One hundred and twenty five μl of isopropyl alcohol was used to precipitate the RNA. The RNA was centrifuged down into a pellet and washed with 75% ethanol two times. The alcohol was discarded and the RNA pellet was dried and resuspended in molecular grade water. RNA concentration was measured (Take3, BioTek Instruments, Winooski, VT).

RNA was converted to cDNA using the High Capacity RNA-to-cDNA kit™ (Life Technologies). Reverse transcription PCR was performed on 1 μg of total cDNA from each sample for control and HP-BMSCs. Each PCR reaction was performed with a mixture of Taq polymerase (New England Biolabs, Ipswich, MA) and its corresponding 10x Taq buffer (New England Biolabs), forward and reverse primers, 10 mM dNTP, water, and cDNA. In particular, we used primers probing for anti-apoptotic and trophic factors. PCR samples were run out on a 1.8% agarose gel with ethidium bromide and visualized under UV light. A list of the factors that we probed for and their primer pairs are listed below alphabetically. All lane intensities were normalized against the corresponding 18S control. PCR gels were captured with a gel imaging system and levels of intensity were quantified with ImageJ.

Primer pairs (5′->3′):

18S (NCBI Accession: NR_003278):

GACTCAACACGGGAAACCTC (forward), ATGCCAGAGTCTCGTTCGTT (reverse)

Bcl-xL (NCBI Accession: L35049):

GCTGGGACACTTTTGTGGAT (forward), CAGTGTCTGGTCACTTCCGA (reverse)

CXCR4 (NCBI Accession: NM_022205):

GCCATGGCTGACTGGTACTT (forward), CACCCACATAGACGGCCTTT (reverse)

FAK (NCBI Accession: JN971016.1):

CAGGGTCCGACTGGAAACC (forward), GTTACTTCCTCGCTGCTGGT (reverse)

HIF-1α (NCBI Accession: AF057308.1):

TGGTCAGCTGTGGAATCCA (forward), GCAGCAGGAATTG (reverse)

SDF-1α (NCBI Accession: BC006640):

GCTCTGCATCAGTGACGGTA (forward), CCAGGTACTCTTGGATCCAC (reverse)

VEGF (NCBI Accession: NM_001287056.1):

CTCACCAAAGCCAGCACATA (forward), AAATGCTTTCTCCGCTCTGA (reverse).

### Focal ischemia stroke model of the adult mouse

All animal experiments and surgical procedures were approved by the Emory University Animal Research Committee and met NIH standards. The animal protocol (2001290-021015BN) specifies the housing location of animals in the temperature and huminity controlled rooms in the Emory University animal facility. The justification of using the mouse stroke model and the number of animals were provided in the protocol. The sterile method, surgery proceudres, pre-surgery and post-surgery procedures are specified. The animal monitoring methods for anesthesia, during surgery, after surgery, the sign of pain and infection are specified in the protocol. The post-surgery care including food and water supplies and prevention of pain and infection using antibiotics and analgesic drugs are described. At the endpoint of experiments, animals will be euthanized using overdose isoflorune.

The sensorimotor cortex ischemic stroke was induced based on previous reports [26, 27]. 8–10 week-old adult male C57BL/6 mice from Jackson Laboratories weighing 26–30 g were used in this investigation. The ischemic surgery procedure was performed following our published method [27]. Briefly, anesthesia was induced using 3.5% isoflurane followed by the maintenance dose of 1.5% isoflurane. Both the tail and paws of the animal were pinch-tested for anesthetic depth. The right middle cerebral artery (MCA) was permanently ligated using a 10-0 suture (Surgical Specialties Co., Reading, PA), accompanied by a bilateral common carotid artery (CCA) ligation for 10 min. This ischemic procedure was suitable and sufficient for the induction of focal ischemia in the mouse brain, resulting in specific infarct formation in the right sensorimotor cortex [27]. Body temperature remained at 37 °C using a heating pad and a temperature-controlled incubator. Three days after stroke, all mice received 50 mg/kg daily BrdU injection i.p. until they were euthanized Fig. 1).

### Intranasal administration of BMSCs

BMSCs were treated with HP as described above. Prior to transplantation, HP-BMSCs were incubated in Hoechst 33342 (1:10,000) for 1 h during reoxygenation. The cells were rinsed with PBS and dissociated from the dish with 0.25% Trypsin–EDTA. 15% FBS growth medium was added to inactivate the trypsin and the cell suspension was collected and centrifuged at 1000×*g* for 3 min, the media was removed, and cells were resuspended at approximately 1 × 10^6^ cells/100 μl. Three, 4, 5, and 6 days after stroke and 30 min prior to BMSC administration, each mouse received a total of 10 μl (10 mg/ml) hyaluronidase (Sigma, St. Louis, MO; dissolved in sterile PBS) delivered into the nasal cavity (5 μl in each nostril). Hyaluronidase increases tissue permeability of the nasopharyngeal mucosa that facilitates stem cell invasion into the brain [28]. One set of animals was randomly designated as the control group receiving cell culture media (100 μl total/animal) and the other set was given BMSCs (approximately 1 × 10^6^ cells/100 μl). Rat cells were used in this experiment due to the greater yield of cells from rats compared to mice. Five drops containing control media or cell suspension were pipetted in each nostril, alternating each nostril with 1-min intervals.

### Tracking BMSCs after transplantation

Six and 24 h after intranasal administration of BMSC, mice were anesthetized with 4% chloral hydrate (10 ml/kg, i.p.) and euthanized once deemed non-responsive. Their brains were dissected out, flattened for tissue sectioning tangential to the surface of the cortex, and mounted in Optimal Cutting Temperature (OCT) compound (Sakura Finetek USA Inc., Torrence, CA, USA) on dry ice. Tissues were sectioned at 10 μm thickness and counterstained with propidium iodide (PI) for nuclear label. Co-labeling of Hoescht 33342 dye positive cells with PI counterstain verified true nuclear labeling of BMSCs in the brain. The peri-infarct area of the cortex was examined for transplanted BMSCs.

### Immunohistochemistry and quantification

Immunohistochemistry was performed to analyze neurogenesis and angiogenesis in vivo. Design-based stereology was used when sectioning fresh frozen brains coronally at 10 μm thickness on a cryostat (CM 1950, Leica Biosystems, Buffalo Grove, IL). Every tenth section was collected such that two adjacent tissues were at least 100 μm apart to avoid counting the same cell twice during analysis. Tissues were collected to include the peri-infarct and infarct areas 1 mm anterior and 1 mm posterior to bregma.

Brain sections were dehydrated on a slide warmer for 15 min and fixed with 10% buffered formalin for 10 min. The sections were washed with PBS (1×, pH 7.4) three times and fixed with methanol twice for 7 min each. Slides were air-dried for several seconds then rehydrated in PBS. Sections were incubated in 2 N HCl for 1 h at 37 °C and then washed in borate buffer for 10 min. Tissue sections were permeabilized with 0.2% Triton X-100 for 45 min and washed in PBS three times. Brain sections were blocked with 1% cold fish gelatin (Sigma) and incubated overnight at 4 °C with the following primary antibodies: Ms anti-NeuN (1:200; MAB377, Millipore, Billerica, MA), Rat anti-BrdU (1:400; AbD Serotec, Hercules, CA), and Rabbit anti-Glut-1 (Chemicon Millipore). Slides were then incubated for 1 h at room temperature with the following secondary antibodies: BrdU: Cy3 anti-rat (1:300, Jackon ImmunoResearch); NeuN: anti-Mouse (1:100, Alexa Fluor 488, Life Technologies, Grand Island, NY); and Glut-1 Cy5 anti-Rabbit. Slides were mounted with Vectashield mounting media and cover-slipped and stored at − 20 °C.

Brain sections were imaged under fluorescent microscopy. Six fields per section were photographed at 40x magnification of both sides of the peri-infarct area in the cortex. Six tissue sections of per animal were photographed. The numbers of BrdU/NeuN co-labeled cells and BrdU/Glut-1 co-labeled cells were quantified using the Image J software (NIH). The reported number for each animal is the sum number of NeuN/BrdU and Glut-1/BrdU co-labeled cells in the image sampling.

### Local cerebral flood flow (LCBF) measurement

Animals were anesthetized with 4% chloral hydrate (10 ml/kg, i.p.). The skin was incised to expose the skull over the peri-infarct area for blood flow measurement. Laser scanning imaging was performed by Laser Doppler flowmetry [29] (PeriFlux System 5000-PF5010 LDPM unit, Perimed, Stockholm, Sweden) and used to estimate the LCBF. The laser was placed to scan above the right MCA and blood flow was measured by the LDPI program. Blood flow was recorded at the same location at the stroke penumbra before stroke, during, and 21 days after stroke. Values were averaged from 6 repeated readings from each time point for both BMSC and control mice. Quantification of the post-stroke mean intensity values were normalized to pre-stroke mean values to measure the change in blood flow over time.

### Adhesive removal functional behavior test

The adhesive removal test was used to evaluate the animal’s sensorimotor impairment after an ischemic stroke as previously described [30]. The stroke affects the forepaws somatosensory cortex thus affecting forepaw sensation in this focal ischemia model. Briefly, an adhesive sticker (Tough-Spots 3/8″ diameter, Diversified BioTech, Dedham, MA) was cut into quarters and placed on one paw of the animal. The time required for the animal to remove the adhesive sticker was recorded in seconds. All mice were trained for 3 days before stroke with one trial run per day to ensure that animals were able to remove the adhesive sticker. Animals were then tested 3 days before, 7, and 14 days after ischemia. The time was averaged from 4 trials per animal. Stereotypically, theunimpaired right paw (contralateral to the unimpaired cortex) has a quicker detection and removal time compared to an impaired left paw (contralateral to the impaired cortex).

### Statistical analysis

Data was graphed and statistically analyzed using GraphPad Prism, version 4 (GraphPad Software, Inc., San Diego, CA). Data analysis was performed using a student’s two-tailed t test for the comparison of the two experimental groups (hypoxic preconditioning PCR, angiogenesis, and neurogenesis data) and two-way ANOVA for multiple comparisons with Bonferroni post hoc tests (Laser Doppler blood flow and adhesive removal test). Changes were identified as significant if p was less than 0.05.

## Results

### Regenerative factors expressed in BMSCs

**Fig. 2:**
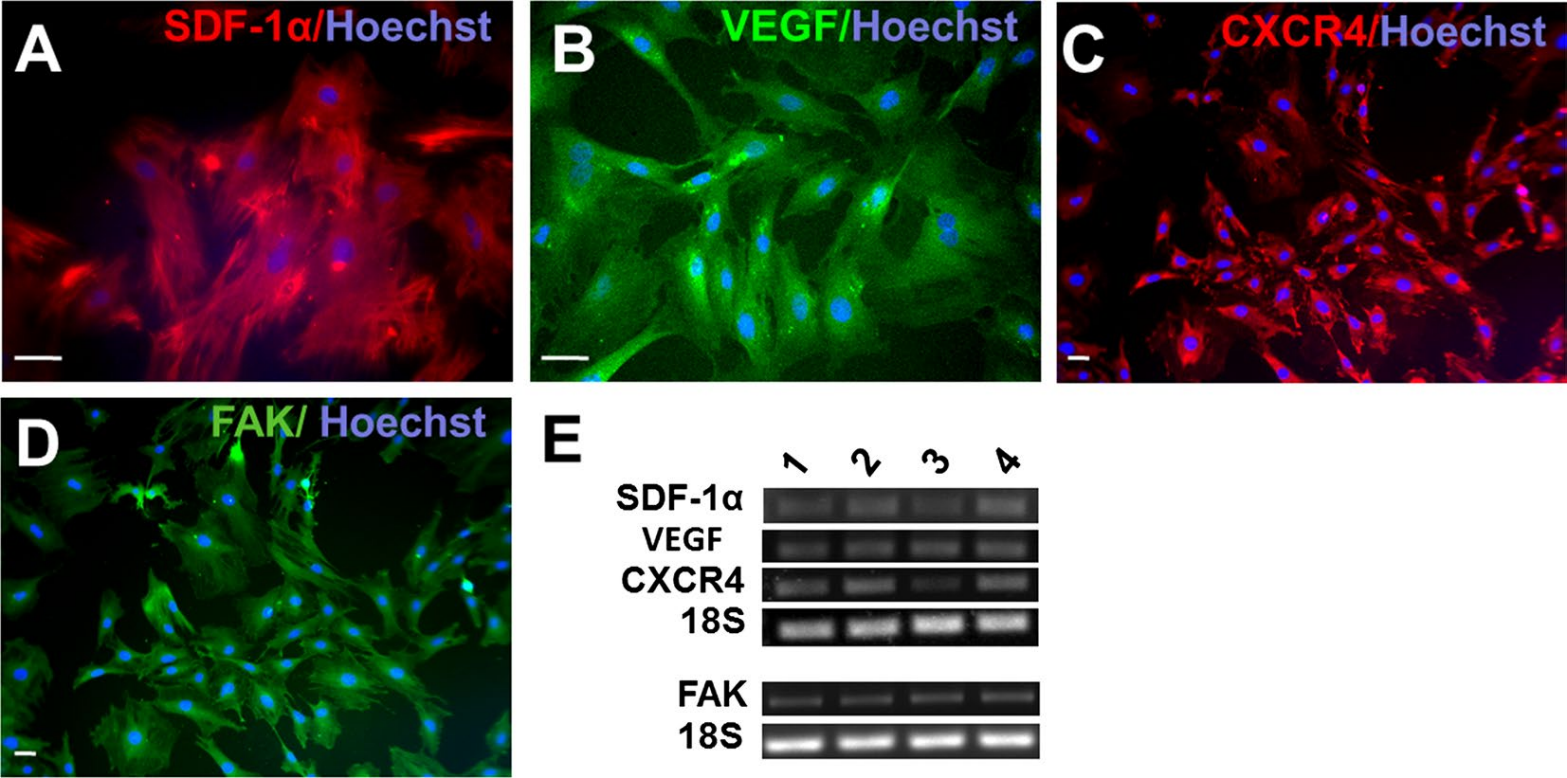
Regenerative factors expressed in BMSCs. Immunocytochemistry was performed in cultured BSMCs for the expression of several regenerative factors. **A**–**D** Expressions of SDF-1α, VEGF, CXCR4, and FAK were detected in BMSCs. Scale bars represents 20 μm. **E** Reverse transcription PCR analysis confirms the expression of SDF-1α, VEGF, CXCR4, and FAK in four different batch of BMSCs

Immunocytochemical staining (Fig. 2A–E) and reverse transcriptase PCR analysis (Fig. 2F) revealed that rat BMSCs in cultures expressed several regenerative and migration factors such as SDF-1α, VEGF, CXCR4 and FAK.

### Hypoxic-preconditioning increases survival factors in BMSCs

**Fig. 3:**
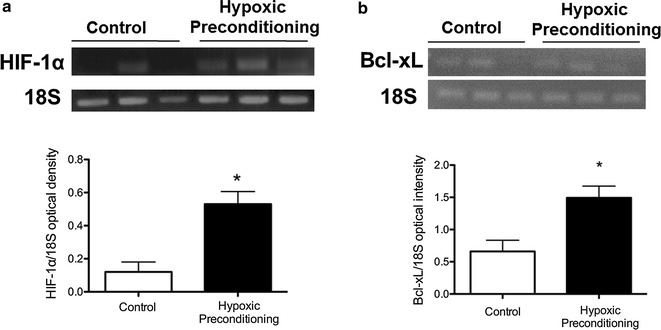
Hypoxic-preconditioning increases survival factors in BMSCs. BMSCs were subjected to 24 h hypoxic preconditioning (0.1–0.3% oxygen) and 1 h of reoxygenation. RT-PCR analysis was used to detect the mRNA level of two key genes for cell survival, HIF-1α and Bcl-xL. **a** HIF-1α in HP-BMSCs was increased compared to control BMSCs. N = 3, p = 0.013; student’s *t* test. **b** Increased Bcl-xL after HP treatment compared to control BMSCs. N = 3, *p < 0.05, student’s *t* test

To enhance the survival and regenerative potential of BMSCs, cultured BMSCs were subjected to 24-h hypoxic preconditioning (HP) treatment in a hypoxic chamber of 0.1–0.3% oxygen. To verify that BMSCs were responsive to hypoxia, RT-PCR showed that the HIF-1α expression was drastically higher after HP (Fig. 3a). Bcl-xl is a prominent downstream factor of HIF-1α [31]. The Bcl-xl level was also significantly increased in BMSCs treated with HP compared to control (Fig. 3b).

### Intranasally delivered BMSCs migrated to the peri-infarct region of the ischemic brain

**Fig. 4:**
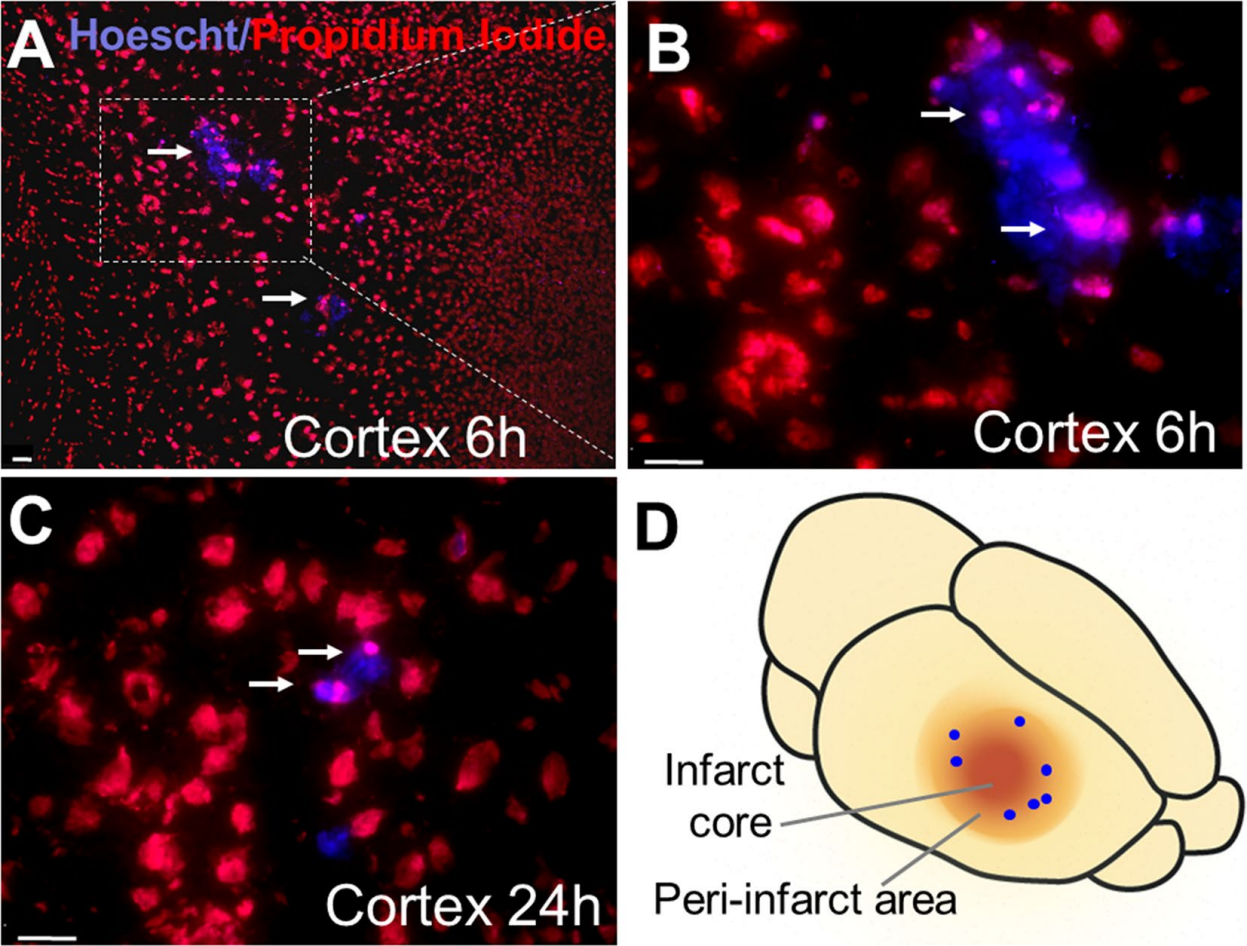
Intranasally delivered BMSCs migrated to the peri-infarct region of the ischemic brain. **A**–**C** Brain sections at the stroke penumbra region were collected and analyzed to examine cell presence of BMSCs delivered intranasally. Hoescht-positive BMSCs (blue) counterstained with Propidium Iodide (PI, red) were detected in the peri-infarct area of the cortex at 6 and 24 h after BMSC delivery at 3 days after stroke. Scale bars = 20 μm. **D** An illustration of the mouse ischemic brain where the cells were found in the cortex

In a mouse model of focal ischemic stroke targeting the right sensorimotor cortex, HP treated rat BMSCs (HP-BMSCs; 1 × 10^6^ cells/100 μl) were intranasally administered at 3, 4, 5, and 6 days after stroke. Transplanted BMSCs were pre-labeled with Hoechst 33342 for in vivo tracking. Due to the well-established and overwhelming benefits of HP, we opted to only use preconditioned cells in this investigation [3]. BMSCs labeled with Hoechst 33342 were detected in the peri-infarct region 6 and 24 h after single BMSC administrations (Fig. 4).

### Intranasally delivered BMSC increased neurogenesis and angiogenesis in the ischemic brain

**Fig. 5:**
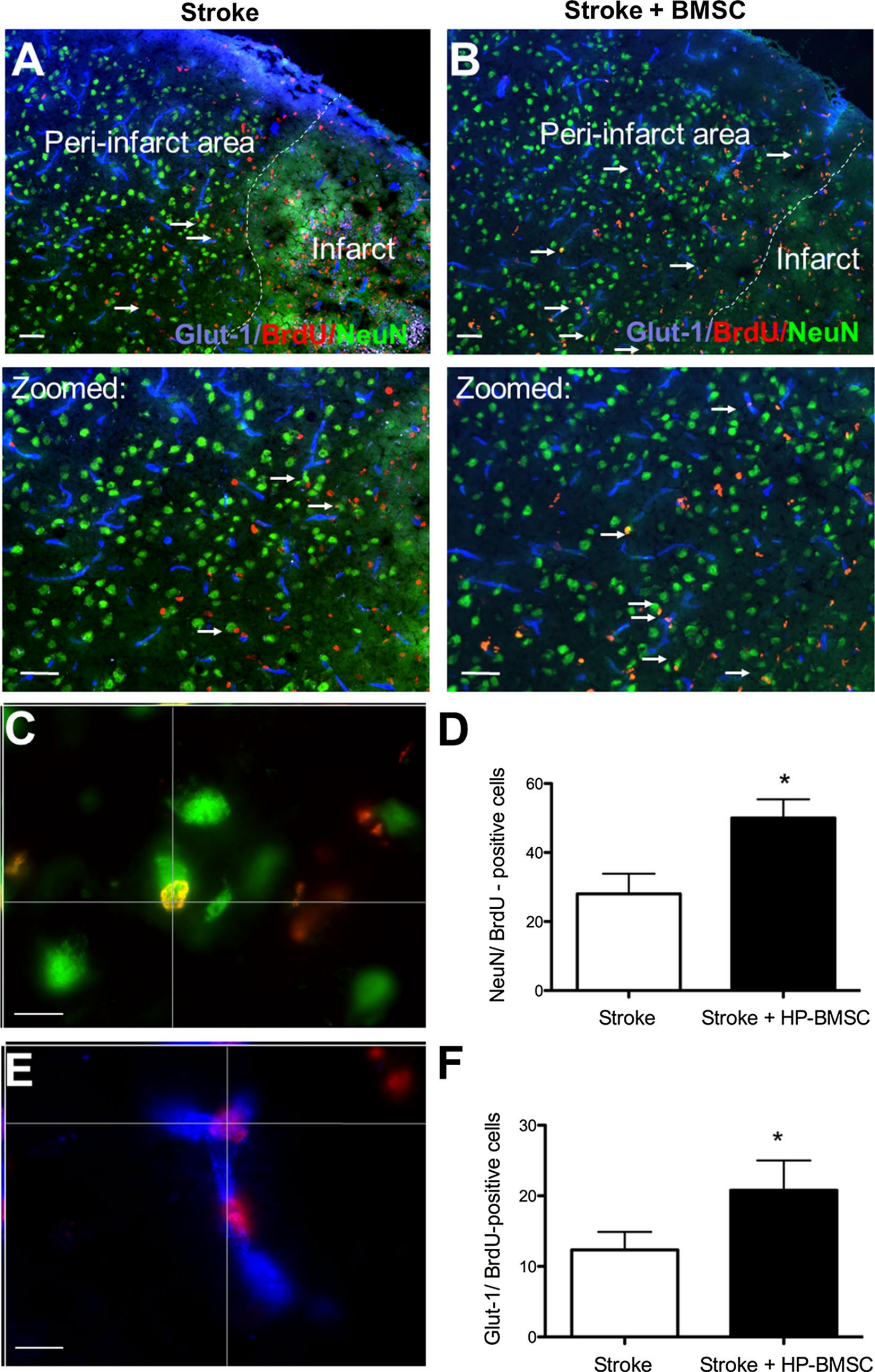
Intranasally delivered BMSC increased neurogenesis and angiogenesis in the ischemic Brain. **A**, **B** Animals were euthanized 14 days after stroke with and without BMSC treatment. Immunohistochemistry stained for BrdU (red), NeuN (green), Glut-1 (blue). Arrows point to the presence of co-labeled cells in the peri-infarct region 14 days post stroke. BrdU/NeuN co-labeled cells indicate the presence of proliferating neuronal cells. BrdU/Glut-1 co-labeled cells indicate the presence of proliferating blood vessel cells. Scale bars = 40 μm. **C**, **D** Enlarged image to show a colabeled NeuN and BrdU cell and the counting result of these cells is shown in the bar graph. There was a significant increase in the total number of NeuN/BrdU co-labeled cells in the BMSC treatment group compared to control. N = 5–6, p = 0.024; student’s *t* test. Scale bar = 10 μm. **E**, **F** Enlarged image shows Glut-1 and BrdU double positive endothelia cells. There was an increase in Glut-1/BrdU co-labeled cells in the HP-BMSC treatment compared to control. N = 5–6, *p < 0.05, student’s *t* test. Scale bar = 10 μm

To assess neurovascular regeneration, we quantified NeuN/BrdU and Glut-1/BrdU co-labeled cells in the peri-infarct area of each animal (Fig. 5). In the peri-infarct cortex 21 days after stroke (18 days after the first delivery of BMSCs), there was a significant increase in NeuN/BrdU co-labeled cells (Fig. 5C, D) and Glut-1/BrdU co-labeled cells (Fig. 5E, F) compared to vehicle-treated stroke controls.

### BMSC transplantation increased local cerebral blood flow (LCBF)

**Fig. 6:**
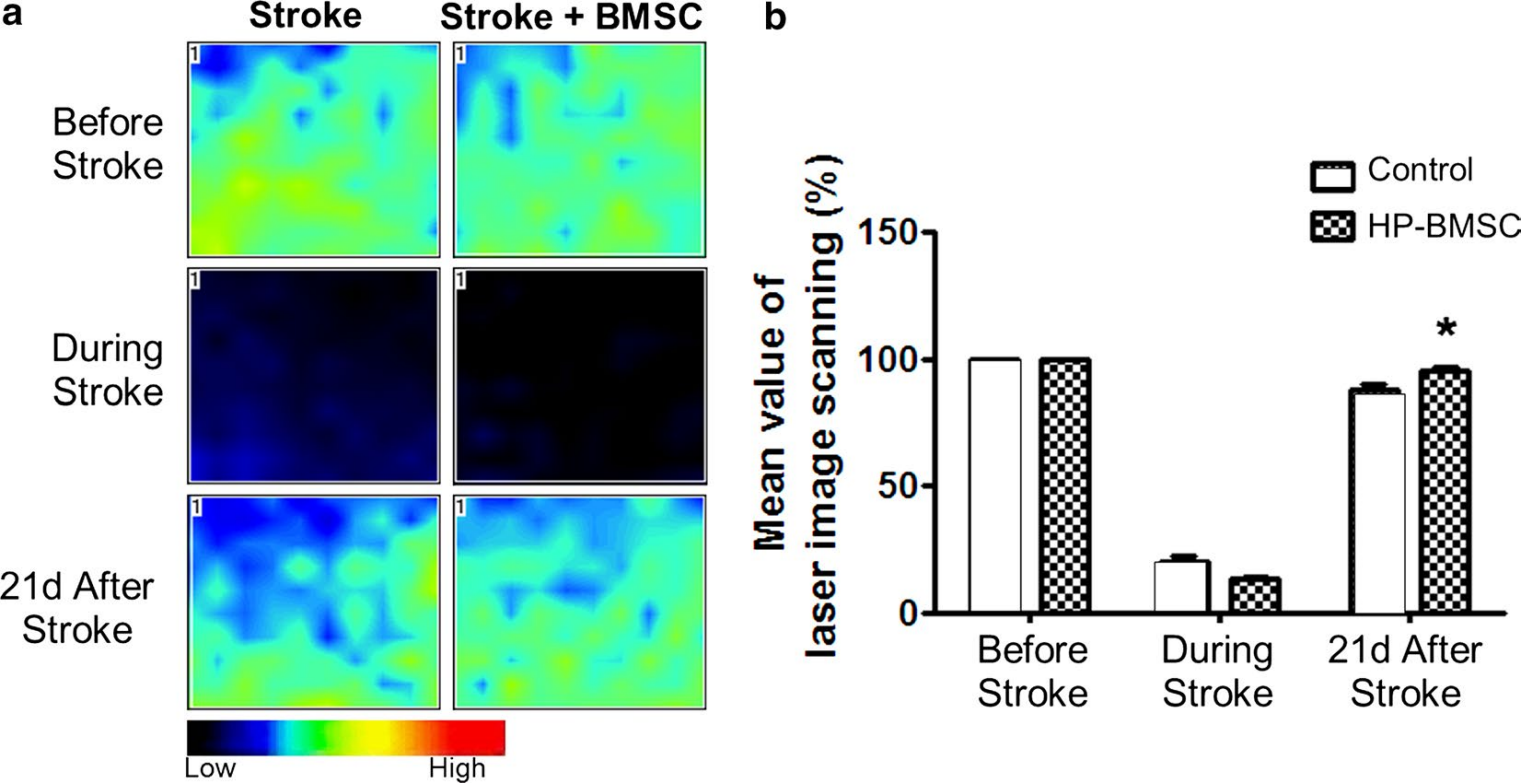
BMSC transplantation increased local cerebral blood flow. Laser-Doppler blood perfusion monitor (PeriFlux System 5000-PF5010 LDPM unit, Perimed, Stockholm, Sweden) was used to measure changes of local cerebral blood flow in the penumbra. **a** Laser scanned images of the stroke penumbra before, during and 21 days after cerebral ischemia in control and BMSC treatment groups. **b** Quantified data of flow measurement 21 days after stroke show similar flow reduction during the ischemic surgery but better restoration of the local cerebral blood flow in BMSC treatment animals compared to controls. N = 5–10, *p < 0.05, two-way ANOVA

The laser Doppler scanning method was used to survey the LCBF at the stroke peri-infarct region before, during, and 21 days after focal stroke [29]. The measurement was taking at the same location over the right somatosensory cortex bordering the ischemic core (Fig. 6a). The mean post-stroke blood flow value was normalized to the mean pre-stroke value. There was a significant increase in LCBF in the BMSC treatment group compared to vehicle controls (Fig. 6b).

### BMSC transplantation increased functional recovery after stroke

**Fig. 7:**
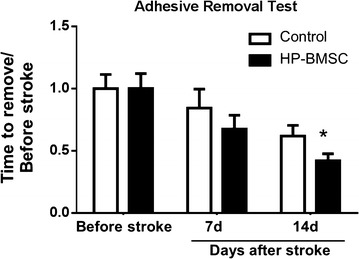
BMSC transplantation increased functional recovery after stroke. The adhesive removal test was used to assess the sensorimotor impairment after ischemic stroke. The test on both control and treatment groups was performed at 3 days before stroke, and 7 and 14 days after stroke. The BMSC treatment group at 14 days displayed significant improvement (shorter time) to remove the sticky dot compared to control mice. N = 8, *p = 0.0503, two-way ANOVA

The adhesive removal test was used to assess sensorimotor deficits in mice 7 and 14 days after stroke induction. In the focal stroke model with damaged right somatosensory cortex, the left paw of the animal is affected. We tested both left and right paws for comparisons. The data was then normalized to the pre-stroke baseline of each individual mouse to account for the natural variation of their removal times before injury. The BMSC treatment group showed significant reduction in removal time of the left paw at 14 days compared with vehicle-treated mice (Fig. 7).

## Discussion

In the present investigation, we demonstrated that delayed multiple intranasal deliveries of BMSCs showed improved vascular and neural regeneration and functional recovery in a focal ischemic stroke model of adult mouse. We confirmed previous findings that BMSCs express several trophic and migratory factors including SDF-1α, CXCR4, VEGF and FAK [24]. These factors play prominent roles in cellular migration, neurogenesis, and angiogenesis [15, 24, 32, 33]. It is predicted that the BMSCs acted as carriers to deliver these factors to the ischemic brain for recovery. As a strategy to enhance the endogenous repair potential of these cells, hypoxic preconditioning was applied as a routine pre-treatment of transplanted cells [3, 34]. Our present study further confirms that HP treatment effectively increases the expression of several pro-survival and pro-regenerative factors in BMSCs, which prime them for greater therapeutic benefits after transplantation.

To be successful in a regenerative therapy, several obstacles must be overcome. One of the major issues of stem cell transplantation after stroke is cell survival after engraftment. Previous studies report that the survival rate of transplanted cells is low, most cells die within 3 days of engraftment likely due to the cytotoxicity from the ischemic environment [35–37]. The timing of stem cell administration may impact graft survival. To maximize transplant efficacy, delayed administration of BMSCs until after levels of inflammatory cytokines have significantly subsided is a feasible approach [38]. To increase the tolerate of transplanted cells to the harsh ischemic environment of the post-stroke brain, we have shown before and in the current investigation that preconditioning BMSCs with hypoxia before transplantation is an effective strategy to increase the expression of specific gene transcripts that are adaptive to low oxygen conditions [39, 40].

The intranasal route can be used to non-invasively deliver neuropeptides and drugs to the brain, but is relatively a new method for cell treatment of stroke [41–43]. It was necessary to demonstrate the feasibility and efficacy of delayed and repeated administration of BMSCs. Intranasally delivered cells can bypass the BBB at the nasal mucosa, pass through the nasal epithelium, and enter the brain via olfactory sensory nerves, such as the trigeminal nerve [28, 42]. It is also possible that intranasally delivered cells can gain access to the CSF and perivascular spaces, further facilitating their transport into deeper brain regions. In the present study, intranasally delivered BMSCs reached the peri-infarct region as early as 6 h after delivery. This is consistent with our previous report showing the ability for the cells to quickly migrate to the brain parenchyma within 1.5 h after intranasal delivery [3].

The homing mechanisms of the SDF-1α/CXCR4 axis and FAK pathways are known to contribute to the migration of HP-BMSCs [3, 44]. SDF-1α is endogenously upregulated in the brain after ischemic injury, forming a chemoattractive gradient that is strongest in the core starting at 7 days after stroke [17]. HP of BMSCs increases CXCR4 expression, suggesting an important role of the SDF-1α/CXCR4 axis in HP-mediated homing of HP-BMSCs to the stroke region [3, 26]. We demonstrate that CXCR4 is expressed in BMSCs, thus these cells can respond to SDF-1α signaling for directed migration toward the lesion site. A previous study of BMSCs injected into the retro-orbital venous sinus of stroke animals revealed that BMSCs migrated to ischemic region closely associating with reactive astrocytes and vessels expressing SDF-1α [44].

FAK is an essential downstream signaling partner in the CXCR4 signaling cascade. FAK phosphorylation can increase cellular migration. We demonstrated before that HP increased BMSC homing to the stroke site was mediated by the mechanism involving FAK and CXCR4 upregulation [3]. FAK is a crucial protein kinase integrating extracellular signaling and cellular migration [45]. HP increases total FAK levels as well as the activated form of phosphorylated FAK [24]. Taken together, our previous and current data indicate that HP promotes the migration mechanism that allows efficient homing of intranasally delivered BMSCs to the peri-infarct region.

Our goal was to test whether or not delayed and repeated administration of BMSCs could increase regenerative activities after stroke. In the current study, we observed that BMSC transplantation exhibits a number of beneficial effects to the injured brain [3, 46, 47]. We found significant increases in neurogenesis and angiogenesis in the peri-infarct area 14 days after stroke in animals that received intranasal BMSCs. It is thought that the trophic factors expressed by BMSCs played a role in neurogenesis. Transplantation of BMSCs with SDF-1α released into this area may attract more endogenous neural progenitors to the peri-infarct area. VEGF is a major trophic factor for the stimulation of angiogenesis. For example, VEGF increased tubule formation and vessel growth of human endothelial cells (HUVEC) in vitro [48]. In stem cell transplantation, neural stem cells that secrete VEGF increased the neovascularization and overall blood vessel density in the peri-infarct area [49]. Further, neural stem cell transplantation enhances angiogenic pathways with increased levels of VEGF and its ligands [49]. VEGF can be neuroprotective and neurogenic. For example, VEGF was shown to promote neurogenesis in the SVZ and SGZ and endogenous migration of neural progenitors from the SVZ [50]. Mice over-expressing VEGF had fewer neurological deficits and smaller infarct volumes after a stroke [51]. Release of VEGF by intranasally administered HP-BMSCs may play a similar role in facilitating angiogenesis.

The neural-vascular interaction in the neurovascular niche plays a major role for functional recovery after stroke [52]. New neurons must interact with the vasculature for the remodeling process and ultimately for animal functional recovery. We tested the effect of intranasally delivered BMSC on blood flow recovery by measuring cerebral blood flow in the peri-infarct area. Mice that received BMSCs had increased local cerebral blood flow 21 days after stroke indicative of vascular improvement. This is consistent with the increased angiogenic activity in BMSC-treated mice at 14 days after stroke.

Our stroke model creates an infarct in the forepaw somatosensory area, thus forepaw somatosensation deficits can be evaluated with the adhesive removal behavioral assay. By measuring the animals’ time to remove the adhesive from the paw contralateral to the ischemic cortex, we assessed functional improvements after the intranasal BMSC therapy. Animals received BMSCs had quicker removal times of the adhesive dot compared to controls. These behavioral data are consistent with the observed increase in neurogenesis, angiogenesis, and local cerebral blood flow. It is postulated that both neurogenesis and angiogenesis worked synergistically to rebuild the neurovascular unit to allow for sustained improvements in somatosensory function.

## Conclusions

We demonstrate the success of repeated administrations of BMSCs, which could increase the number of stem cells delivered to the target site. The time for the regenerative treatment can be delayed beyond 24 h after stroke, which is a clinically relevant therapeutic window for most stroke patients.

## Abbreviations

BDNF: brain-derived neurotrophic factor
BMSCs: bone marrow stromal cells
BrdU: bromodeoxyuridine (5-bromo-2′-deoxyuridine)
CCA: common carotid artery
CNS: central nerves system
CXCR4: C-X-C chemokine receptor type 4
FAK: focal adhesion kinase
Glut-1: glucose transporter 1
HP: hypoxic preconditioning
HIF-1α: hypoxia induced factor-α
LCBF: local cerebral blood flow
MCA: middle cerebral artery
OCT: optimal cutting temperature
PBS: phosphate buffered saline
RT-PCR: reverse transcription polymerase chain reaction
SDF-1α: stromal cell-derived factor-1α
SVZ: subventricular zone
tPA: tissue plasminogen activator
VEGF: vascular endothelial growth factor
